# Bacterial profile and antibacterial susceptibility of otitis media among pediatric patients in Hawassa, Southern Ethiopia: cross-sectional study

**DOI:** 10.1186/s12887-019-1781-3

**Published:** 2019-11-01

**Authors:** Bereket Tadesse, Techalew Shimelis, Mesfin Worku

**Affiliations:** 1 Hawassa University College of Medicine and Health Science, Comprehensive Specialized Laboratory, Hawassa, Ethiopia; 2 Hawassa University College of Medicine and Health Science, Hawassa, Ethiopia; 3 College of Medicine and Health Science, School of Medical Laboratory, Hawassa university, Hawassa, Ethiopia

**Keywords:** Paediatric ear infection, Bacterial isolates, Antibiotic resistance

## Abstract

**Background:**

Otitis Media (OM) is the most common disease of childhood. Twenty thousand people die each year from otitis media. It is an important cause of preventable hearing loss, affects children's intellectual performance and language development. There are very small numbers of studies done in Ethiopia concerning this topic. This study aimed to identify bacterial pathogens related to ear infection and to assess antibacterial susceptibility of isolated organisms.

**Method:**

A cross-sectional study was conducted on 152 children from April 2018 to July 2018 at selected health facilities in Hawassa city, SNNPR, Ethiopia. All pediatric patients having ear discharge were included. Convenient sampling technique was used to collect clinical and demographic data using standard questionnaires after child care-takers signed the consent. Ear discharge specimens were collected using a sterile swab, and transported using Amies transport media to Hawassa University Comprehensive Specialized Hospital laboratory. Bacterial isolates were characterized based on colony appearance, Gram reaction, culture characteristics, and biochemical tests after inoculating on appropriate culture media. Antibacterial susceptibility testing was performed using the disc diffusion method according to the criteria of the Clinical and Laboratory Standards Institute (CLSI).

**Results:**

Among 152 children included, 115(75.6%) of them demonstrated pathogenic bacterial growth. *Staphylococcus aureus* 41(27%) was the most frequently isolated pathogen, followed by *Proteus mirabilis* 19 (12.5%). Of the total isolates, 11.2 and 7.3% were resistant to gentamicin and ciprofloxacin respectively. Over three-fourth (85.2%) of the isolates were resistant to ampicillin. More than two-third of the isolates were resistant to both penicillin (71.4%) and trimethoprim-sulphamethoxazole (72.0%).

**Conclusions:**

*S. aureus* is the most commonly isolated bacterial pathogen from ear discharge among children. Even though gentamicin is a parenteral drug and ciprofloxacin is rarely used in children due to concerns of bone/joint effects, these two drugs were highly effective antibiotics and thus should be considered in treating children with otitis media since most organisms were resistance or poor response to first line drugsHigh level of antibiotic resistance was observed so antimicrobial susceptibility test is needed before prescribing drugs for treatment of OM.

## Background

Otitis Media is the most common disease of childhood, with about 65–330 million people suffering from ear infection worldwide and 60% of them have significant hearing impairment [1]. It is estimated that 20,000 people die each year from otitis media and the overall burden of these diseases is higher in the poorest countries [2]. The health-economic burden of ear infection is also severe especially in Africa and other developing nations where the disease prevalence is estimated as high as 11% [3].

Complications of Otitis Media (OM) such as perforation of the tympanic membrane, otitis externa and mastoiditis affect balance, motor control and hearing. Acquired hearing impairment in children is mainly caused by chronic suppurative otitis media (CSOM). The hearing impairment produced by otitis media also affect intellectual performance and language development [4, 5].

The growing bacterial resistance to antibiotics has altered the clinical picture of otitis media and its complications. The emergence of Multi Drug Resistance (MDR) is clearly related to the dose of antibiotics and how they are being used [6]. Serious infections caused by methicillin-resistant *S. aureus* (MRSA) are increasingly difficult to treat. While it is clear that antibiotics are pivotal in the selection of bacterial resistance, the spread of resistance genes and resistant bacteria also contributes to the problem [7]. Most of the time treatment for OM is done clinically, especially in health facilities found in low income countries. This will cause bacterial drug resistance and preventable complications of OM such as deafness and meningitis [8] .

There are a small number of studies done in Ethiopia concerning this topic, moreover, almost all of them show the emergence of these drug resistant bacterial strains and recommend further study [9] . Therefore this study aimed to identify bacterial pathogens related to ear infection and to assess antibacterial resistance among isolated organisms.

## Methods

### Description of the study area and period

Hawassa is the capital of the Southern Nations, Nationalities and People's Region and is located 275km from Addis Ababa, the capital city of Ethiopia. The study was conducted in the Hawasa University Comprehensive Specialized Hospital, Adare Hospital, Tilte Health Centre and Rama Paediatric Clinic. Those sites are selected based on reports of higher rates of otitis media in children (personal communication with health facilities and by reviewing the 6 month report). This facility based cross- sectional study was conducted from April 2018 to July 2018.

#### Sample size

A total of 152 study participants were included using single population proportion formula based on estimated prevalence (p) taken from Yitayal S. et al. in 2013 which was 90% in pediatric patients, margin of error (5%), 95% confidence level CI (z = 1.96).

### Data collection

#### Demographic characteristics and risk factors

Data on socio-demography and potential risk factors were collected using a questionnaire developed for this study. Data collectors (a translator as necessary) approached children with ear discharge at the outpatient department of the study sites and provided information for the parents or care-takers regarding the study. After allowing them to think and discuss and attending all questions, they were asked to sign the informed consent. Those patients whose care takers signed the written consent were interviewed by health officers or a physician. All paediatric patients under the age of 15 years with ear discharge were included whereas children on antibiotic treatment in last 2 weeks were excluded.

#### Specimen collection, transport and processing

Ear discharge specimens were collected at collection sites aseptically by an ENT Specialist or health officers using a sterile cotton swabs after the patient's ear was washed by normal saline (0.85% NaCl). Samples were kept in Amies transport media to maintain the viability of microorganisms until the specimen is processed. And specimens were transported for processing within 2h to Microbiology laboratory of Hawassa University Comprehensive Specialize Hospital (HUCSH).

#### Sample processing and culturing

All ear discharge specimens were inoculated on blood agar, chocolate agar, and MacConkey agar. All inoculated agar plates were incubated at aerobic atmosphere whereas chocolate agar was incubated in carbon dioxide enriched atmosphere using a candle jar to enhance the growth of *S. pneumoniae* at 37°C for 24–48h [10] . Preliminary identification of all positive cultures was done based on observation of growth on primary culture media and examination of Gram-staining reaction under microscope.

#### Characterization of isolated bacteria

Bacterial isolates were characterized based on colony appearance, gram reaction, culture characteristics, and biochemical tests as described by Cheesbrough. Biochemical tests carried out include: catalase, urease, coagulase, oxidase, carbohydrate fermentation, motility, gas production, H_2_S production, citrate utilization, and Indole test. Satellitism test and XV factor tests was done for identification of *H. influenza.* Identification of *S. pneumonae* and *Entrococcus spp* was done using Optochin disc test [10] . Identification of MRSA was done using cefoxitin disc diffusion method and MLSBi was identified by performing the D test. *Staphylococcus aureus* isolates showing circular zones of inhibition with diameter of ≤13mm for ERY and ≥21mm for CLN without a D-shaped zone along the ERY were interpreted as negative for inducible resistance (D-test negative). *S. aureus* isolates with same inhibitory diameters as above with a D-shaped zone around the CLN were interpreted as positive for inducible resistance (D-test positive) [11].

#### Antibacterial susceptibility testing

Antibacterial susceptibility testing was performed by disc diffusion method for all isolates according to the criteria of the Clinical and Laboratory Standards Institute (CLSI) version 2017. From a pure culture 3–5 selected colonies of bacteria were taken and transferred to a tube containing 5â€‰ml nutrient broth and mixed gently until a homogenous suspension was formed and incubated at 37°C until the turbidity of the suspension become adjusted to a McFarland 0.5. A sterile cotton swab was used and the excess suspension was removed by gentile rotation of the swab against the internal surface of the tube. The swab was then used to distribute the bacteria evenly over the entire surface of Mullen Hinton agar. The inoculated plates were left at room temperature to dry for 3 to 5â€‰min and a set of antibiotic discs such as ampicilin (10g), ciprofloxacilin (5μg), gentamicin (10μg), ceftriaxone, penicilin G (10μg), cefoxitin, amoxicillin-clavulonate (30μg), chloroamphenicol, and tetracycline (30μg) were dispensed on the surface of the inoculated Muller-Hinton plate. All drugs were selected based on recommendation of CLSI guidelines (2017 version). For fastidious organisms, a chocolate agar plate (for *H. infuenzae*) and modified Muller-Hinton agar (for *S. pneumoniae)* were used. The inoculated plate was incubated at 37°C for 18-24h [11].

#### Data management and quality control

The questionnaire was first prepared in English and was translated into Amharic and back translated into English by another person to check its consistency. Before the actual data collection time, the questionnaire was pre-tested using 26 volunteers (5% of the sample), in Hawassa University's Comprehensive Specialized Hospital to check for any missing options, ambiguity and clarity. Data quality was ensured through use of standardized data collection materials. Proper training was given for data collectors before the start of data collection and intensive supervision was done during data collection by the principal investigator.

For laboratory analysis pre-analytical, analytical and post-analytical stages of quality assurance in standard operating procedures (SOPs) of the microbiology laboratory were strictly followed. In addition, well-trained and experienced laboratory professionals participated in the laboratory analysis procedure. Quality control strains such as *E. coli* (ATCC-25922), *S. aureus* (ATCC-25923) and *P. aeruginosa* (ATCC-27853) were included from the Armauer Hansen research institute (AHRI) to check all media and biochemical tests and sensitivity. Performance tests were also conducted on 5% of prepared media.

#### Data analysis

Data collected with questionnaires and laboratory investigations were entered and analyzed using SPSS version 20 (IBM Corp. Released 2011. IBM SPSS Statistics for Windows, Version 20.0. Armonk, NY: IBM Corp.). Study findings were presented in words and tables. Frequencies and proportions were used to summarize socio-demographic and other factors. Multivariable logistic regression analysis was performed taking those socio-demographic and risk factors found to be significantly associated with bacterial ear infection in bivariate logistic regression analysis. Associations between dependent and independent variables were assessed and strength was described using odds ratios and 95% confidence intervals. *P*-values less than 0.05 were interpreted as statistically significant.

## Results

### Description of socio demographic characteristics

A total of 152 children with ear discharge were included in this study from April 2018 to July 2018. Among these, 78/152 (51.3%) were males. The age range of participants was from 3months to 12years, with a mean age of 2.59 years. Nearly half of participants 80/152 (52.6%) were in the age group of 1 to 4years, and the majority of the study subjects 105 (69.5%) were urban residents [Table 1].

**Table 1:** Socio demographic characteristics of children with ear discharge, Hawassa, SNNPR, April to July, 2018

Variables		Number	Percent
Sex	Male	78	51.3
	Female	74	48.7
Age (in years)	< 1	48	31.6
	1–4	80	52.6
	5–10	21	13.8
	11–15	3	2.0
Residence	Urban	105	69.5
	Rural	47	30.9
Place of stay in day time	Home	118	77.8
	School	32	21.1
	Day care	2	1.3

### Culture results

Among 152 individuals whose samples were cultured, at least one pathogenic bacterial isolate were detected in 115/152 (75.7%). A total of 123 bacterial isolates were detected; of which 59/123 (48.0%) were Gram positive and 64 (52.0%) were Gram negative bacteria. Eight samples had two different types of bacterial isolates.

*Staphylococcus aureus* 41/152 (27.0%) was the most frequently isolated pathogen followed by *P. mirabilis* 19/152 (12.5%)*, Haemophilus influenzae* 14/152 (9.2%), *E. coli* 11/152 (7.2%), *Enterococcus species* 10/152 (6.6%), *Streptococcus pneumoniae* 8/152 (5.3%), *Klebsiella pneumoniae* 6/152 (3.9%), and *Klebsiella ozeanae* 6/152 (3.9%). The majority of the bacterial isolates 99/152 (65.1%) were found in children less than 5 years of age [Table 2].

**Table 2:** Bacterial isolates among age groups of study participant with ear infection visiting selected health facilities in Hawasa, Ethiopia, April 2018 July 2018

Isolated organism	Age group of the study participant			Total(*n*= 152)
	< 1 year (*n* =48)	1 years (*n* =80)	6–12years (*n*= 24)	
*S. aureus*	14	17	10	41 (27%)
*P. mirabilis*	1	16	2	19 (12.5%)
*H. influenzae*	12	2	0	14 (9.2%)
*E. coli*	5	6	0	11 (7.2%)
*Enterococcus species*	4	4	2	10 (6.6%)
*S. pneumoniae*	6	2	0	8 (5.3%)
*K .pneumoniae*	0	6	0	6 (3.9%)
*K. ozienae*	0	6	0	6 (3.9%)
*Pseudomonas species*	1	3	0	4 (2.6%)
*Providenica species*	0	0	2	2 (1.3%)
*Acinetobacter species.*	0	2	0	2 (1.3%)
*Total*	37 (24.3%)	62 (40.8)	16 (10.5)	152 (100%)

*n* number of participant

Twelve out of 14 *H. influenza* isolates were identified from the children less than 12â€‰months of age. On the other hand, out of total 19 *P. mirabilis* isolates, 16/19 (84.2%) were identified from children aged between 1 and 5 years. Moreover, *S. aureus* had higher frequency in all age groups. The frequency of *Pseudomonas species* was only 4 (2.6%).

### Drug resistance patterns

*E. coli* (36.4%) and *Enterococcus species* (50.0%)showed resistance to ciprofloxacin but the rest of all isolated Gram positive and Gram negative bacteria showed no (0.0%) resistance to ciprofloxacin. On the other hand, all Gram negative bacteria except *P. mirabilis* (68.4%) were 100% resistant to ampicilin. 11.2% of pathogens showed resistance to gentamycin but only *K. pneumonia* was 100% resistant. *Klebsiella species* showed higher resistance for most of the antimicrobials tested, but isolates were susceptible to ciprofloxacin [Table. 3].

**Table 3:** Antimicrobial resistance patterns of Gram-negative bacteria isolated from ear infection among children visiting selected health facilities, Hawassa, Ethiopia, 2018

Isolated organisms	AMP	CIP	SXT	GEN	AUG	CTR	CAZ	MER	CHL	TET
*P.mirabilis*	68.4%	0.0%	57.9%	10.5%	57.9%	10.5%	10.5%	21.1%	31.6%	ND
*E.coli*	100%	36.4	72.7%	18.2%	81.8%	18.2%	18.2%	0.0%	18.2%	ND
*H.influenza*	100%	0.0%	16.7%	ND	50%	50%	66.7%	100%	0.0%	ND
*K.oziena*	100%	0.0%	66.7%	33.3	66.7%	33.3%	33.3	33.3%	33.3%	33.3%
*K.pneumonia*	100%	0.0%	100%	100%	66.7%	100%	66.7	33.3%	66.7%	66.7%
*Pseudomonas.spp.*	ND	0.0%	ND	25	ND	100%	25	25.0%	ND	ND
*Acinetobacter spp*	100%	0.0%	100%	0.0%	100%	100%	100%	0.0%	0.0%	100%
*Providenicaspp*	100%	0.0%	100%	0.0%	50%	100%	50%	0.0%	0.0%	100%

KEY; *AMP* Ampicilin, *CIP* Ciprofloxacin, *SXT* Trimethoprim-sulphamethoxazole *GEN* Gentamycin, *AMC* Amoxicillin Clavulanic acid, *CTR* Ceftraxone, *CAZ* Ceftazidime, *MER* Meropenem, *CHL* Chloraphinicol, *TET* Tetracyclin, *ND* not done

*S. pneumonia* showed no resistance for any of the drugs tested. *S. aureus* showed higher resistance to penicillin (85.4%) and trimethoprim-sulphamethoxazole (73.2%). However, all isolates of *S. aureus* were susceptible to ciprofloxacin and gentamycin. Only half of the isolated *Enterococcus species* were susceptible to both ciprofloxacin and erythromycin. All *Enterococcus species* are susceptible to chloraphenicol [Table.4].

**Table 4:** Antimicrobial resistance patterns of Gram-positive bacteria isolated from ear infection among children visiting selected health facilities, Hawasa, Ethiopia, April 2018 to July

Isolated organisms	CIP	GEN	SXT	PEN	CLN	CHL	CXT	TET	ERY
*S.aureus*	0.0%	0.0%	73.2%	85.4%	14.6%	14.6%	17.1%	24.4%	14.6%
*S.pneumonia*	ND	ND	ND	ND	0.0%	0.0%	ND	ND	0.0%
*Enterococcus spp.*	50%	ND	ND	37.3	ND	0	ND	37.30%	50.0%

KEY; *CIP* ciprofloxacin, *GEN* Gentamycin, *SXT* Trimethoprim-sulphamethoxazole, *PEN* Penicillin, *CLN* Clindamycin, *CHL* Chloraphenicol, *CXT* Cefoxitin, *TET* Tetracycline, *ERY* Erythromycin, *ND* not done

Regarding drug resistance rates, 85.2, 72.0 and 71.4% of the isolated bacteria were resistant to ampicilin, trimethoprim-sulphamethoxazole and penicillin respectively. These were followed by 37.5, 31.3, 22.6 and 11.2% of the isolates resistant to ceftriaxone, ceftazidime, chloramphenicol and gentamicin respectively. Only 7.3% of the isolates were resistant to ciprofloxacin.

From the total (41) isolated *S. aureus*, 6 (14.6%) showed erythromycin induced clindamycin resistance also known as MLSBi resistance which is observed as a â€˜Dâ€™ shape on Muller Hinton agar and 7(17.0%) of them were methicilin resistant *S. aureus* (MRSA). Out of the seven MRSA identified, 2(28.5%) of them showed erythromycin-inducible clindamycin resistance.

### Description of potential risk factors

From the 152 study subjects 52(34.4%) of them had an ear infection longer than 2 weeks and 57 (37.5%) of the participants had a history of taking antibiotics. Eighty six (56.6%) of the children included in this study were breast feeding and 56 (36.8%) of the children were fed by bottles. Upper respiratory tract infection, including throat infection, is known to be a risk factor for otitis media. From all children included this study, 89(58.9%) had a URTI and 32 (20.4%) had a throat infection. Among all study subjects, 20 (13.2%) came from households with tobacco smokers, and 10 (6.6%) of the patients were observed to have another person having an ear infection in their family and 57(37.5%) of them were taking antibiotics [Table 5].

**Table 5:** Frequency and percentage of possible risk factors for bacterial ear infection of children with ear discharge, Hawassa, SNNPR, April 2018 to July 2018

Variables		Frequency	Percent
Duration of infection	Less than 2 weeks	100	65.8
	More than 2 weeks	52	34.4
Breast feeding	Yes	86	56.6
	No	66	43.4
Bottle feeding	Yes	56	36.8
	No	96	63.2
Presence of throat infection	Yes	31	20.4
	No	121	79.6
Presence of tobacco smoke in the house	Yes	20	13.2
	No	132	86.8
Presence of URTI	Yes	89	58.6
	No	63	41.4
Other ear infection in the family	Yes	5	3.2
	No	147	96.7
Antibiotic treatment history	Yes	57	37.5
	No	95	62.5

*URTI* Upper Respiratory Tract Infection

### Association of the potential risk factors and culture positive otitis media

Factors associated with detection of pathogenic bacteria from ear discharge in children have been analyzed but there was no statistically significant association between detection of pathogenic bacteria and most potential risk factors. However, the presence of upper respiratory tract infection showed a statistically significant relationship, with an odds ratio of 4.5 (95% CI 1.8–12.0). Bottle feeding happened to have a negative effect on the detection of pathogenic bacteria from ear discharge, with an odds ratio of 6.5 (95% CI 2.4–17.8) [Table 6].

**Table 6:** Factors associated with detection of pathogenic bacteria from ear discharge among children at selected health facilities in Hawasa,Ethiopia April 2018 to July 2018

Characteristics		Number tested	Culture positive	COR	*P*-value	AOR	*P*-value
			N (%)	(95%CI)		(95%CI)	
Sex	Male	78	59 (75.6)	1 (0.5–2.1)	0.99	0.54 (0.2–1.5)	0.19
	Female	74	56 (75.7)	1	–	1	–
Age group	≤5	128	99 (77.3)	1.7 (0.6–4.3)	0.26	4.1 (1–15.8)	0.38
	> 5	24	16 (66.7)	1	–	1	–
URTI	Yes	89	74 (83.1)	2.6 (1.2–5.6)	0.012	4.5 (1.8–12)	0.001
	No	63	41 (65.1)	1	–	1	–
Throat infection	Yes	31	23 (74.2)	0.9 (0.36–2.2)	0.83	0.8 (0.3–2.4)	0.79
	No	121	92 (76.0)	1	–	1	–
Bottle feeding	Yes	56	35 (62.5)	1	–	1	–
	No	96	80 (83.3)	3 (1.4–6.4)	0.005	6.5 (2.4–17.8)	0.000
Breast feeding	Yes	86	64 (74.4)	1.1 (0.55–2.5)	0.68	0.4 (0.1–1.2)	0.11
	No	66	51 (77.7)	1	–	1	–
Duration of infection	≤ 15 day	100	77 (77.0)	0.8 (0.4–1.7)	0.59	2.0 (0.8–5.1)	0.11
	>15day	52	38 (73.1)	1	–	1	–
Residence	Urban	105	77 (73.3)	0.65 (0.3–1.5)	0.32	0.55 (0.19–1.5)	0.26
	Rural	47	38 (80.9)	1	–	1	–
Antibiotic treatment	Yes	57	43 (75.4)	0.98 (0.4–2.1)	0.96	1.4 (0.6–3.4)	0.42
	No	95	72 (75.8)	1	–	1	–

*COR* Crud odds ratio, AOR- Adjusted odds ratio

## Discussion

Otitis media is a major health problem in children in developing countries [12]. OM is more common in children due to the fact that children's eustachian tube is shorter, more horizontal and with a more flaccid cartilage which can easily impair its opening and leads to ear infection [4, 12]. Most cases of OM arise between the age of 6 month and 3 years [13].

In this study, we have been able to see the etiological agents and antimicrobial resistance pattern of isolates from children with ear infection visiting health facilities in Hawassa from April to July 2018. The overall rate of ear infection with positive culture results was 75.6%, which is higher than finding in Ethiopia (48.5%) [14] but comparable with other parts of the world including Nigeria (91%) and Yemen (78.0%) [8, 15] . The probable reasons for this could be attributed to difference in study participant, study period, climate and geographical variation.

In the present study, a similar percentage of Gram negative and Gram positive bacteria were detected (52.0 and 48.0%, respectively), which is in agreement with an earlier report in Gonder (56.0 and 44.0%) [16] .This finding was different from a study done in Hawasa (79.5%) [17]. Possible reasons for this difference could be age group dissimilarity and study period.

The finding of this study showed that *S. aureus* was the most prevalent (27.0%) pathogen among children having ear discharge, which was similar with the previous studies done in our country [14, 17] and abroad in Nepal [18, 19]. However, the result was in contrast to other reports such as a study from Bahirdar Regional Health Research Laboratory Centre [16] and in other parts of the world, *Pseudomonas* species is the most frequently isolated organism [2]. The probable reason could be that the frequency of *S. aureus* in the middle ear infection could be attributed to their ubiquitous nature and high carriage of resistant strains in the external auditory canal and upper respiratory tract.

In the present study, the prevalence of *Pseudomonas* species was 4 (2.6%). However, study conducted around the world reported *Pseudomonas* spp. as the most frequently isolated Gram negative pathogen from ear discharge [8] . The possible reason is that most study subjects included in this study had OM which is less than 2 weeks. Apparently, *Pseudomonas* species is mostly isolated from chronic OM, which is more than 2 weeks of duration or more. The other reason for this fact could be that the time or season of a year may favour some type of bacteria [15].

Our study indicates that the majority of the bacterial isolates (65.1%) were found in children less than 5 years of age. A similar finding was also documented in some previous studies done in Gonder University Hospital, and Ayder Teaching and Referral Hospital, Mekele [9, 20]. Twelve out of 14 *H. influenza* isolates were identified from children less than 12 months of age. This is due to the fact that the main bacterial isolates in AOM are the same as those that typically infect the upper respiratory tract in children and *H. influenza* is known to cause URTI [20]. Out of *P. mirabilis* isolates, 84.2%were identified from children aged between 1 and 5 years. Similar results were reported from a study done at EPHI [14].

In this study, lower resistance was observed to ciprofloxacin (7.3%) and gentamicin (11.2%), though resistance rates were high to ampicilin (85.2%), trimethoprim-sulphamethoxazole (72.0%)and penicillin (71.4%), which was consistent with several reports elsewhere [4, 8, 16, 18]. *Klebsiella* species are known for their resistance to several drugs [10], and a similar finding was also observed in the current study. *S. aureus* showed higher resistance for penicillin and trimethoprim-sulphamethoxazole with 85.4 and 73.2%, respectively. This is comparable with the study done in Jimma, where 84% of *S. aureus* isolates were found to be resistant to penicillin [19] . Even though gentamicin is a parenteral drug and ciprofloxacin is rarely used in children due to concerns of bone/joint effects, these two drugs were highly effective antibiotics and thus should be considered in treating children with otitis media since most organisms were resistance or poor response to first line drugs. Gentamicin should not be used as mono-therapy for *S. aureus* infections. Indeed, there is little evidence it has any utility in any *S. aureus* infections. *S. aureus* showed no resistance to ciprofloxacin and gentamicin which is in agreement with studies done in Jimma and Bahirdar [16, 19] . The probable reasons for this variation could be attributed to antimicrobial resistance profile of bacteria varies among population because of difference in geography, local antimicrobial prescribing practices and prevalence of resistant bacterial strains.

Factors associated with detection of pathogenic bacteria from ear discharge in children have been analyzed but there was no statistically significant association between detection of pathogenic bacteria and most of the potential risk factors. However, presence of upper respiratory tract infection showed a statistically significant relation, with an AOR of 4.5 (95% CI 1.8–2.0). This was in agreement with the result in Jimma [19]. It is also known that upper respiratory tract infections are one of the major reasons for impaired middle ear physiology [13].

## Conclusion

Otitis media is still a major health problem of children and bacterial agents are the main cause. The most commonly isolated bacterial pathogen from ear discharge among children is *S. aureus* followed by *P. mirabilis*. There was a high level of antibiotic resistance observed among isolates of ear discharge from children. Ampicilin, penicillin and trimethoprim-sulphamethoxazole were less effective against the isolates. Apparently, ciprofloxacin, gentamicin and chloraphinecol showed good effect against most bacterial pathogens that cause OM. Drug resistant strains of *S. aureus* such as MRSA and MSLBi can cause ear infection. Upper respiratory tract infection is a risk factor for ear infection among children.

## Data Availability

If someone wants to request the data, it is available on hand of Bereket Tadesse.

## Abbreviations

AOM: Acute Otitis Media
BAP: Blood Agar Plate
CAP: Chocolate Agar Plate
CLSI: Clinical and Laboratory Standards Institute
CSOM: Chronic Suppurative Otitis Media
EPHI: Ethiopian Public Health Institute
HUCSH: Hawassa University Comprehensive Specialized Hospital
MAC: Macckonkey Agar
MDR: Multi Drug Resistance
OM: Otitis Media
OME: Otitis Media with Effusion
PBP: Penicillin Binding Protein
SNNPR: Southern Nations Nationalities People Region
URTI: Upper Respiratory Tract Infection
WHO: World Health Organization
